# Surgical success in obstetric fistula repair and associated factors: findings from a retrospective cohort study in Zambia

**DOI:** 10.1186/s12893-025-02910-z

**Published:** 2025-04-17

**Authors:** Sianga Mutola, Bwalya Magawa Chomba, Nawi Ng, Dhally M. Menda, Valérie R. Louis, Lowery Wilson Michael

**Affiliations:** 1 Faculty of Medicine and University Hospital, Heidelberg Institute of Global Health, Heidelberg University, Heidelberg, Germany; 2 Fistula Foundation Treatment Network in Zambia, Plot 001 Samfya Road, Mansa, Zambia; 3 School of Public Health and Community Medicine, Institute of Medicine, University of Gothenburg, Sahlgrenska Academy, Gothenburg, Sweden; 4 School of Postgraduate Studies, University of Lusaka, Lusaka, Zambia; 5 Injury Epidemiology and Prevention (IEP) Research Group, Turku University Hospital and University of Turku, Turku, Finland

**Keywords:** Obstetric fistula, Obstetric care, Fistula repair, Surgery, Zambia

## Abstract

**Background:**

Obstetric fistulas are common in low-resourced settings, but the factors associated with successful repair remain unclear in Zambia. We assessed the socio-demographics, fistula characteristics, and healthcare factors associated with successful obstetric fistula repair outcomes in Zambia.

**Methods:**

Our retrospective cohort study was based on the Zambia Fistula Foundation Treatment Network’s clinical database, including 1439 women who underwent obstetric fistula surgical repairs at hospitals in Zambia between 2017 and 2023. Tanahashi’s Health Services Coverage framework guided the selection of potential factors associated with successful obstetric fistula repair outcomes. We employed Multivariate Imputation by Chained Equations (MICE) before conducting logistic regression analyses. Univariable models, a multivariable model, and marginal probabilities were then fitted to examine the associations between successful obstetric fistula repair outcome, a fistula that is closed and dry, and relevant covariates.

**Results:**

Our results showed an overall fistula repair success rate of 88.1%. Patients from the Northern and Muchinga provinces showed 51% (AOR = 0.49, 95% CI = 0.27, 0.90) lower odds of surgical repair success compared to those from Central and Lusaka provinces. Patients with a previous fistula repair had 47% lower odds of success (AOR = 0.53, 95% CI = 0.32, 0.87) than those without. Finally, surgeries rated intermediate in difficulty had 64% (AOR = 0.36, 95% CI = 0.18, 0.70), and those rated difficult had 90% (AOR = 0.10, 95% CI = 0.05, 0.21) lower odds of success than simple repairs.

**Conclusion:**

We identified geographic location, previous repair history, and surgical complexity as the factors associated with successful obstetric fistula repair outcomes.

## Introduction

Obstetric fistulas are a common adverse outcome of prolonged obstructed labor in resource-limited settings, including sub-Saharan Africa [1–3]. An obstetric fistula injury occurs during prolonged obstructed labor when the fetal head compresses the birth canal tissues, bladder base, urethra, or rectum, causing tissue death and creating an abnormal hole between the vagina and bladder (vesicovaginal) or between the vagina and rectum (rectovaginal) [2, 4]. Women who experience prolonged obstructed labor often face restricted access to comprehensive emergency obstetric care, including emergency cesarean sections, which are critical interventions for preventing further complications such as the formation of obstetric fistulas [5]. Obstetric fistulas and other poor outcomes of prolonged obstructed labor have been substantially minimized in most high-income countries [3, 6]. However, they continue to be a notable public health concern in resource-constrained settings, specifically in sub-Saharan Africa, mainly due to limited access to quality maternal health services, stunting, and harmful traditional practices like early marriages and teenage pregnancies [7–11]. It is estimated that 2 million women live with obstetric fistulas globally [12], with an obstetric fistula population-based prevalence rate of 1.60 cases per 1,000 women aged 15 to 49 years in sub-Saharan Africa (SSA) [8, 12].

Obstetric fistulas are preventable through improving access to perinatal healthcare services such as antenatal care, promotion of institutional delivery, and comprehensive emergency obstetric care, including timely caesarian sections in the event of obstructed labor [3]. Furthermore, obstetric fistulas can be repaired, in most cases, by surgery, and interventions have been implemented to prevent and repair fistulas by governments and cooperating partners such as the Fistula Foundation Treatment Network and the United Nations Population Fund – UNFPA [9, 13]. However, many women still face barriers in accessing surgical repairs in a context of few surgeons trained by the International Federation of Gynecology and Obstetrics (FIGO), a limited number of accessible health facilities that offer fistula repair surgeries, myths, and misconceptions about obstetric fistulas, and social and gender norms that might delay decision-making to seek care [1, 5, 7, 14]. The barriers and other harmful factors experienced by women as they access obstetric fistula surgical treatment might lead to inequalities in the outcomes of the surgical repairs [5, 15, 16]. Since joining the UNFPA-led Global Campaign to End Fistula in 2005, Zambia has expanded its capacity for fistula repair, with nearly 2,000 surgeries performed over the past decade [7, 17, 18]. The Ministry of Health in Zambia and the Fistula Foundation Treatment Network have supported the training of additional fistula surgeons, conducted fistula repair outreach camps, and provided routine care for fistula patients at hospitals in all the regions of the country [18]. However, the number of specialist fistula surgeons remains too small to handle the overwhelming number of obstetric fistulas in Zambia. For instance, Zambia had only four trained fistula repair surgeons in 2017, thereby limiting rural fistula patients’ access to timely surgeries [18]. The Fistula Foundation Treatment Network has since enhanced surgical capacity by sponsoring the training of additional obstetricians/gynecologists at a FIGO-approved center in Kenya [18]. However, challenges such as high costs, limited resources, and a high backlog of cases hinder comprehensive fistula treatment nationwide [5].

The management of fistula patients includes psychosocial counseling and the preoperative assessment of patients using methylene blue dye (dye test) to confirm the fistula's presence [7, 19]. Surgical repair of urinary fistulas involves circumferential dissection of the vaginal fistula epithelium, mobilization of the bladder, and tension-free closure of the defect using absorbable sutures, with a watertight seal confirmed by a dye test [19]. In more complex fistula cases, additional steps like colostomy or flap creation may precede definitive repair, followed by postoperative catheterization and a methylene blue test to confirm surgical success, defined as complete closure and absence of fecal or urine incontinence, with or without increased intra-abdominal pressure [7, 19, 20]. With a 24.92% pooled prevalence of obstetric fistula repair failure in sub-Saharan Africa, having a previous repair, total urethral damage, and large fistula size are some factors associated with obstetric fistula repair failure [21]. However, research on obstetric fistula in Zambia, as in other low-resource settings, is limited, with most studies being small-scale and descriptive [5, 18]. Therefore, the factors associated with successful fistula repair outcomes in Zambia remain unclear, as no nationwide hospital-based research exists. A study based on the Fistula Foundation Treatment Network database of fistula repair surgeries at 110 hospitals in 27 countries from Asia and Africa estimated the success rate of fistula repairs to be 87% [13]. Another recent study based on a small sample of cases of women who underwent surgical fistula repairs at Zambia’s University Teaching Hospital (UTH) of Lusaka estimated an 83% fistula repair success rate. Still, it did not establish the factors associated with this success rate [22]. Therefore, there is a need for further comprehensive research to assess the factors associated with successful obstetric fistula repair to maximize the success rate.

Our study aimed to identify the factors associated with surgical success in obstetric fistula repair in Zambia based on the Fistula Foundation Treatment Network’s hospital database on fistula repairs in Zambia. We used the Tanahashi Model to identify the socio-demographic, fistula characteristics, and healthcare factors that influence obstetric fistula repair outcomes in Zambia [23]. The Tanahashi Model, which includes evaluating the availability, accessibility, acceptability, contact, and adequate coverage, was highly applicable to our study as it provided a detailed framework for analyzing how various factors influenced access to, utilization of, and the effectiveness of healthcare services [23].

## Methods

### Study design and study site

This retrospective cohort study used the Fistula Foundation Treatment Network’s nationwide clinical database, including 1439 women who underwent obstetric fistula surgical repairs from 2017 to 2023. During this time, the Fistula Foundation Treatment Network supported the Zambian Ministry of Health in conducting fistula repairs in hospitals from eight regions of Zambia: Chilenje Level 1 Hospital (Lusaka province), Monze Mission Hospital (Southern province), Kabwe Central Hospital (Central province), Solwezi General Hospital (North-western province), St. Francis Mission Hospital (Eastern province), Mbala General Hospital (Northern province), Mansa General Hospital (Luapula province), and Chilonga Mission Hospital (Muchinga province). The women who sought treatment at these hospitals came from across the ten provinces of Zambia [18].

### Data collection

The Zambia Fistula Foundation Treatment Network progressively implemented outreach programs that strengthened the linkages for fistula patients from the communities to the treatment centers where they accessed quality fistula care, including surgical repairs. These community outreach strategies helped to improve awareness, create demand, and enhance patient referral through well-coordinated structures at Primary Health Care (PHC) levels, involving a team of community-based volunteers (CBVs), including Safe Motherhood Action Groups (SMAGs) members and Community Health Workers. The CBVs were drawn from the PHC structures such as Neighborhood Health Committees (NHCs), Health Posts (HPs), and Rural Health Centers (RHCs) and trained on the Ministry of Health’s basic safe motherhood package and the comprehensive fistula continuum of care comprising prevention, care, treatment, and rehabilitation and social reintegration of patients. The CBVs leveraged their rapport with members of their communities by providing accurate information to dispel any myths and misconceptions related to obstetric fistulas and facilitating timely referrals for treatment and care. To refer suspected fistula patients for medical assessments at the treatment centers, the CBVs administered in the community, the verbal Obstetric Fistula Community Based Assessment Tool (O-COMBAT) to identify the signs and symptoms of fistulas. Some patients sought medical care independently after gaining awareness of the services from the radio, through a former client, or a relative. Healthcare providers and the Fistula Foundation staff also directly engaged and referred some fistula patients during their routine community interactions or other health service provision activities. Once the patient reached the treatment center, their Socio-demographic information, information about the fistula characteristics after a thorough physical examination by a surgeon, including using the Methylene Blue test, and the fistula management data after surgery were recorded into the hospital registers and exported into the Fistula Foundation Treatment Network database [18].

### Ethics approval and consent to participate

Our study used the Fistula Foundation Treatment Network hospital database in Zambia. The database was made up of secondary routine hospital patient records. Therefore, the need to obtain informed consent from the patients to participate in this study was waived by the Ethics Commission of the Medical Faculty at the University of Heidelberg (Ref. No: S- 683/2024) and the ERES Converge Institutional Review Board (IRB) (Ref.No:2024-Jul- 011), that provided ethical clearance for the study. Furthermore, healthcare providers collected patients’ information as part of the hospitals’ routine patient record-keeping in line with the Health Professions Act of 2009. Additionally, the healthcare providers obtained informed consent from the legal parents or guardians of patients aged under 16 years for the collection of medical data, as required by the Health Professions Act of 2009. Furthermore, the Fistula Foundation Treatment Network in Zambia authorized the use of the database for our study. Lastly, datasets used for this study were fully pseudonymized by eliminating any personal identifiers, such as names, and each patient was assigned a Fistula Foundation unique identity (ID) number.

### Measurements

#### Outcome variable

In this study, we used the immediate surgical obstetric fistula repair outcome, assessed at discharge from the treatment center, as the outcome variable. All the patients who underwent obstetric fistula surgical repair were assessed upon discharge from the hospital by the medical team using the Methylene blue test to ascertain the continence status of the fistula. The continence status was recorded as “closed and dry”, “closed with stress incontinence”, and “not closed”. For this study, we generated a binary outcome variable by combining the categories “closed with stress incontinence” and “not closed” to represent an “unsuccessful” repair outcome. The “closed and dry” category represented a “successful” repair outcome.

#### Covariates of obstetric fistula repair outcome

We analyzed the socio-demographics, fistula characteristics, and healthcare factors associated with the successful obstetric fistula surgical repair outcome. In this study, the socio-demographic factors included age group, marital status, parity level (number of live births) [24], and province of residence in Zambia. The patients’ age groups were categorized in years as < 20, 20–34, and ≥ 35. Marital status was categorized into not married, married, and unknown. The parity level was classified as 0, 1–3, and ≥ 4. The hospital database collected information about the Zambian provinces where the patients live, and the responses included all ten provinces of Zambia, namely Eastern, Luapula, Lusaka, Central, Copperbelt, Western, Muchinga, Northern, Northwestern, and Southern. The provinces of Zambia are divided into urban and rural [25]. The urban provinces generally include Lusaka (the Capital city) and the Copperbelt (the mining mainstay). According to the 2018 Zambia Demographic and Health Survey, 46% of the population in urban areas falls into the highest wealth quintile. In contrast, in rural areas, only 3% of the population reaches this level [26]. Furthermore, nearly two-thirds of the rural population is concentrated in the lowest wealth categories, with 33% in the lowest quintile and 31% in the second-lowest [26]. Provincially, Western has the highest proportion of people in the lowest wealth quintile, at 47%, Southern at 11.1%, North-Western at 25.5%, Northern at 39.5%, Muchinga at 38.3%, Luapula at 28.7%, Eastern at 31.3%, and Central at 15.9%, with the lowest being Lusaka at 1.6% and Copperbelt at 2.2%. [26]. Therefore, in this study, we grouped the provinces into geographic clusters representing geographic proximity – hence, patients seeking care from the same fistula treatment centers, similarities in cultures, and potentially experiencing similar socioeconomic challenges, making the analysis more meaningful. In this regard, Eastern and Luapula provinces were left to stand alone. Therefore, we grouped the provinces as follows: Central and Lusaka, Copperbelt and North-Western, Eastern, Luapula, Northern and Muchinga, Southern and Western, and Unknown category for those who did not provide this information.

The fistula characteristics included the obstetric fistula type, the obstetric fistula classification, whether the woman had a previous fistula repair, years lived with the fistula, and the surgery type. The responses to the question about the types of fistulas included rectovaginal fistula (RVF), vesicovaginal fistula (VVF), and both (RVF + VVF). We combined both (RVF + VVF) with the RVF into one category called “RVF or both” because evidence shows that having any of these scenarios resulted in a poorer prognosis compared to only having a VVF [16, 27]. Therefore, this variable was dichotomized into “VVF” and “RVF or both”. The operating surgeons used Waaldijk’s fistula classifications [6], thereby recording the fistula types as follows: Type I: Fistula that involves only the bladder (not affecting the continence mechanism) and is located above the urethra. Type II A(a): Fistula consists of the continence mechanism without significant scarring and does not include the urethra. Type II A(b): Fistula involving the continence mechanism with unilateral ureteric involvement but no considerable scarring. Type II B(a): Fistula with substantial scarring involving the continence mechanism but without ureteric involvement. Type II B(b): Fistula with significant scarring involving the continence mechanism and bilateral ureteric involvement. Type III: Complex fistulas involving the urethra, typically with extensive damage and scarring. The surgical team also asked the women if they had previous fistula repair(s), and the responses were dichotomized into yes and no. We categorized the years the women lived with the obstetric fistula into < 1 year, 1–5 years, and > 5 years. Lastly, the type of surgery that the operating surgeons recorded after fistula repair was related to the type of obstetric fistula, such that we categorized all surgical repairs that involved the urinary tract structures as VVF. In contrast, “Others” included RVF and other types, such as perennial tears.

Regarding the healthcare factors, we evaluated the hospitals of care (treatment centers), surgery difficulty, and the presence of postoperative complications as recorded by the surgical team. The hospitals were grouped into logical categories based on their geographic proximity, as patients in those areas are likely to seek health care from any nearby hospital, regardless of provincial boundaries. Therefore, the hospitals were clustered as follows: Lusaka/Central (Chilenje/Kabwe), Northern region hospitals (Mbala/Mansa/Chilonga), St. Francis, Monze Mission, Solwezi General, and Unknown for those who did not provide the information. The operating surgeons recorded the surgical repair’s difficulty as simple, intermediate, or difficult based on specific factors, including a narrow vaginal caliber (< 2 cm), minimal residual anterior vaginal wall, the need for ureteric stenting, bilateral dense lateral contractures, the use of a combined abdominal and vaginal approach, and the presence of a recto-vesicovaginal fistula [19]. We categorized the presence of postoperative complications as none or with a complication.

### Statistical analysis

**Fig. 1:**
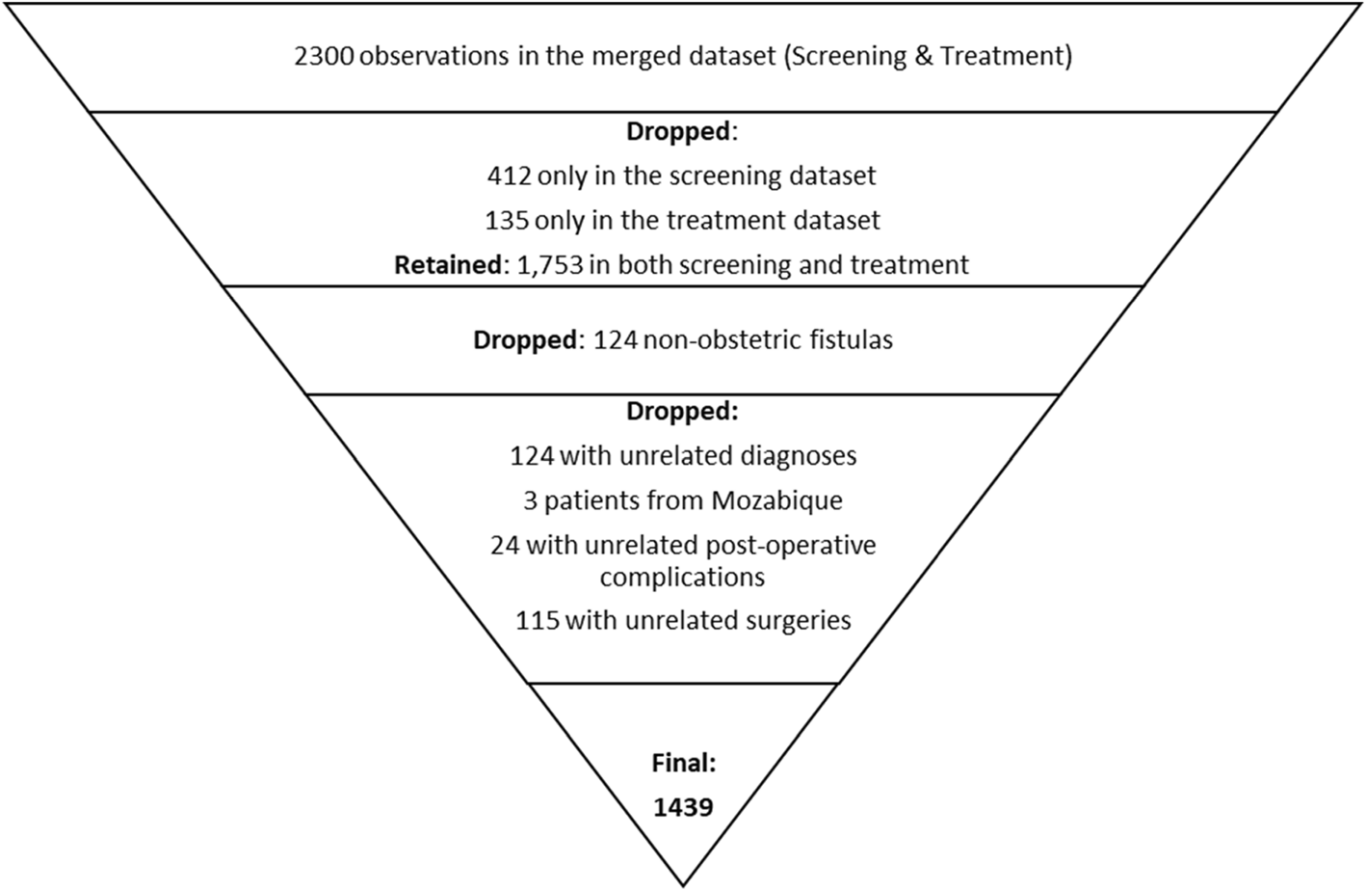
depicts the inclusion and exclusion procedure that we used to retain the appropriate patients in our analysis

The Fistula Foundation Treatment Network database was recorded on two separate Microsoft Excel sheets according to screening and treatment data. The screening dataset included socio-demographic data such as age, marital status, province of residence, and parity. The treatment dataset included the hospital of care and all the fistula and surgery data. In this study, we were interested in evaluating the factors associated with the most recent or last surgical repair outcomes. Therefore, after importing the treatment dataset into Stata, we retained the last treatment and excluded the previous visits before merging it with the screening dataset Fig.1.

After finding no multicollinearities among the variables, we analyzed descriptive statistics from the patients’ socio-demographic information, stratified by the obstetric fistula repair outcome, successful or unsuccessful, reporting row proportions. After checking for missing values, we found that only 41% of the observations had complete data on all the variables. Therefore, we imputed the dataset with 20 imputations by predictive mean matching using Multivariate Imputation by Chained Equations (MICE) before running the logistic regressions [28]. We used the imputed dataset to identify the associations between covariates and successful obstetric fistula repair outcomes in the univariable models and the adjusted model. The univariable models involved a logistic regression for each covariate with the outcome variable, successful obstetric fistula repair outcomes, and results were reported as crude or unadjusted odds ratios (UOR). The adjusted model involved all the covariates added simultaneously in the logistic regression, and results were reported as adjusted odds ratios (AORs). We reported UORs and AORs and their corresponding 95% confidence intervals (95% CIs). After running the adjusted model, we assessed the marginal probabilities for a successful fistula repair outcome for each category of the variables. We used Stata/SE 18 for Windows (StataCorp LLC, 4905 Lakeway Drive, College Station, 77845, USA) to conduct all the analyses.

## Results

### Participants’ socio-demographic information

Table 1 presents the participants’ socio-demographic information, stratified by the obstetric fistula surgical repair outcome, successful or unsuccessful. For instance, our results showed that 88.1% of the fistula repairs were successful, while only 11.9% were unsuccessful at the patient’s discharge from the hospital. Additionally, more than half (53.5%) of the successful surgical repairs were among patients aged between 20 and 34 years, while the majority (54.1%) of those whose surgical repairs were unsuccessful were above 34 years. Regarding marital status, the majority (66.7%) of the women whose surgical repairs were successful were married, compared to only 25.4% who were unmarried. Our results also showed that 44.4% of those whose surgical repairs were unsuccessful were from the Northern and Muchinga provinces, compared to only 0.6% of those from the Southern and Western provinces. The study results also showed that the majority (78.7%) of the women with successful repairs had vesicovaginal fistulas compared to 21.3% who had both or rectovaginal fistulas. Furthermore, 53.2% of the patients whose fistula repairs were unsuccessful had lived with the fistula for more than five years, compared to 12.8% of those who lived with the fistulas for less than a year.

**Table 1:** Characteristics of participants according to the immediate outcome of obstetric fistula repair

	**Not successful**	**Successful**
	**(*N*= 171)**	**(*N* = 1268)**
**Women's age groups**		
< 20	10 (6.8%)	85 (7.1%)
20–34	58 (39.2%)	643 (53.5%)
** ≥ 35**	80 (54.1%)	474 (39.4%)
**Marital Status**		
Not married	60 (35.1%)	322 (25.4%)
Married	89 (52.0%)	846 (66.7%)
Unknown	22 (12.9%)	100 (7.9%)
**Province in Zambia**		
Central + Lusaka	19 (11.1%)	214 (16.9%)
Copperbelt + North-Western	4 (2.3%)	77 (6.1%)
Eastern	23 (13.5%)	254 (20.0%)
Luapula	42 (24.6%)	243 (19.2%)
Northern + Muchinga	76 (44.4%)	396 (31.2%)
Southern + Western	1 (0.6%)	48 (3.8%)
Unknown	6 (3.5%)	36 (2.8%)
**Parity Level**		
**0**	4 (2.4%)	6 (0.5%)
**1–3**	105 (64.0%)	725 (59.8%)
** ≥ 4**	55 (33.5%)	482 (39.7%)
**Fistula Type**		
Rectovaginal Fistula (RVF) or both (RVF + VVF)	11 (6.4%)	266 (21.3%)
Vesicovaginal Fistula (VVF)	160 (93.6%)	984 (78.7%)
**Fistula Classification**		
Type I	29 (23.6%)	251 (33.5%)
Type II A(a)	18 (14.6%)	170 (22.7%)
Type II A(b)	11 (8.9%)	35 (4.7%)
Type II B(a)	30 (24.4%)	92 (12.3%)
Type II B(b)	18 (14.6%)	36 (4.8%)
Type III	17 (13.8%)	165 (22.0%)
**Years with Fistula**		
< 1 Yr	20 (12.8%)	284 (24.5%)
1–5 Yrs	53 (34.0%)	383 (33.0%)
> 5 Yrs	83 (53.2%)	493 (42.5%)
**Had previous repairs**		
No	107 (70.9%)	976 (89.3%)
Yes	44 (29.1%)	117 (10.7%)
**Treatment Hospital**		
Monze and Chilenje	11 (6.4%)	186 (14.7%)
Kabwe and Solwezi	2 (1.2%)	34 (2.7%)
Northern region hospitals	133 (77.8%)	786 (62.0%)
St. Francis	23 (13.5%)	255 (20.1%)
Unknown	2 (1.2%)	7 (0.6%)
**Type of Surgery**		
Others	10 (5.9%)	374 (29.6%)
VVF Repair	160 (94.1%)	890 (70.4%)
**Surgery Difficulty**		
Simple	13 (8.4%)	507 (46.5%)
Intermediate	56 (36.1%)	429 (39.3%)
Difficult	86 (55.5%)	155 (14.2%)
**Post-Operative Complications**		
None	137 (92.6%)	1092 (97.3%)
With complication	11 (7.4%)	30 (2.7%)

### Associations between the covariates and obstetric fistula repair outcome

In our study, we evaluated the factors associated with successful obstetric fistula surgical repair by estimating crude odds ratios and adjusted odds ratios, as shown in Table 2.

**Table 2:** Unadjusted and adjusted associations between immediate successful obstetric fistula repair outcome and covariates

	**Crude (unadjusted) Odds Ratios** **UORs. (*95% CI*)**	**Adjusted Odds Ratios** **AORs. (*95% CI*)**
***Age group***		
< 20	0.79 (0.39, 1.60)	0.69 (0.29, 1.60)
20–34	Ref	Ref
** ≥ 35**	**0.57 (0.40, 0.81)**	0.68 (0.43, 1.07)
***Married***		
Not married	Ref	Ref
Married	**1.77 (1.24, 2.51)**	1.29 (0.85, 1.96)
Unknown	0.84 (0.49, 1.45)	0.80 (0.41, 1.56)
***Parity level***		
**0**	Ref	Ref
**1–3**	**4.43 (1.22, 16.03)**	2.96 (0.50, 17.32)
** ≥ 4**	**5.79 (1.58, 21.25)**	3.71 (0.59, 22.39)
***Province***		
Central & Lusaka	Ref	Ref
Copperbelt & North-Western	1.70 (0.56, 5.18)	2.56 (0.54, 11.99)
Eastern	0.98 (0.51, 1.84)	0.57 (0.23, 1.44)
Luapula	**0.51 (0.28, 0.91)**	0.52 (0.27, 1.02)
Northern & Muchinga	**0.46 (0.27, 0.78)**	**0.49 (0.27, 0.90)**
Southern & Western	4.26 (0.55, 32.63)	3.26 (0.29, 35.91)
Unknown	0.53 (0.19, 1.42)	0.78 (0.27, 2.26)
***Fistula type***		
Rectovaginal Fistula (RVF) or both (RVF + VVF)	Ref	Ref
Vesicovaginal (VVF)	**0.24 (0.13, 0.45)**	2.14 (0.37, 12.31)
***Fistula classification***		
Type I	Ref	Ref
Type II A(a)	1.11 (0.65, 1.91)	1.15 (0.62, 2.13)
Type II A(b)	0.48 (0.23, 1.02)	0.55 (0.23, 1.32)
Type II B(a)	**0.53 (0.31, 0.89)**	0.58 (0.32, 1.06)
Type II B(b)	**0.43 (0.22, 0.82)**	0.62 (0.27, 1.41)
Type III	1.63 (0.93, 2.87)	0.87 (0.44, 1.73)
***Had previous fistula repair***		
No	Ref	Ref
Yes	**0.31 (0.21, 0.47)**	**0.53 (0.32, 0.87)**
***Years lived with fistula***		
< 1 year	Ref	Ref
1–5 years	**0.53 (0.30, 0.90)**	0.54 (0.29, 1.02)
> 5 years	**0.43 (0.26, 0.71)**	0.58 (0.31, 1.09)
***Surgery type***		
Other	Ref	Ref
VVF repair	**0.15 (0.08, 0.29)**	0.22 (0.03, 1.36)
***Hospital of care***		
Monze Mission & Chilenje Level 1	Ref	Ref
Kabwe Central & Solwezi General	1.00 (0.21, 4.74)	0.65 (0.08, 4.96)
Northern region hospitals (Mansa, Mbala & Chilonga)	**0.34 (0.18, 0.65)**	0.91 (0.37, 2.25)
St. Francis Mission	0.65 (0.31, 1.37)	1.33 (0.48, 3.67)
Unknown	0.20 (0.38, 1.11)	0.32 (0.05, 1.79)
***Surgery difficulty***		
Simple	Ref	Ref
Intermediate	**0.22 (0.12, 0.41)**	**0.36 (0.18, 0.70)**
Difficult	**0.60 (0.03, 0.11)**	**0.10 (0.05, 0.21)**
***Post-complication***		
None	Ref	Ref
Yes	**0.36 (0.18, 0.75)**	0.67 (0.30, 1.52)

In the univariable analysis, our results showed that patients aged 35 years or older had 43% (UOR = 0.57; 95% CI = 0.40, 0.81) lower odds of achieving a successful fistula repair than their counterparts aged 20–34 years. Those who were married had 77% (UOR = 1.77; 95% CI = 1.24, 2.51) higher odds of fistula repair success than their unmarried counterparts. Additionally, the patients who had one to three live births and those with four or more live births had 343% (UOR = 4.43; 95% CI = 1.22, 16.03) and 479% (UOR = 5.79; 95% CI = 1.58, 21.25), respectively, higher odds of achieving a successful fistula repair than those who had no live births. Furthermore, the patients from Northern and Muchinga provinces had 51% (UOR = 0.46; 95% CI = 0.27, 0.78) lower odds of fistula repair success than those from Central and Lusaka provinces.

Furthermore, the univariable logistic regression results showed 76% lower odds (UOR = 0.24; 95% CI = 0.13, 0.45) of successful fistula repair outcomes among those who had previous repairs than their counterparts who had none. Similarly, patients who lived with a fistula for 1–5 years had 43% lower odds (UOR = 0.53; 95% CI = 0.30, 0.90), and those who lived with a fistula for over five years had 57% lower odds (UOR = 0.43; 95% CI = 0.26, 0.71) of repair success compared to those who lived with it for less than a year. Furthermore, the patients with a VVF had lower odds of a successful repair outcome than those with other types.

Regarding hospitals of care, our results revealed that patients whose fistulas were repaired at Zambia’s Northern region hospitals (Mansa General, Mbala General, and Chilonga Mission) had 66% lower odds (UOR = 0.34; 95% CI = 0.18, 0.65) of having a successful fistula repair outcome compared to those who were repaired at Monze Mission and Chilenje Level 1 hospitals. As expected, the fistula repair surgeries that were recorded as intermediate or difficult had lower odds of successful repair outcomes than simple ones.

In the adjusted model, the results showed that obstetric fistula patients from Northern and Muchinga had 51% (AOR = 0.49, 95% CI = 0.27, 0.90) lower odds of successful surgical repair compared to those from Central and Lusaka provinces. Furthermore, the patients who had a previous fistula repair showed 47% (AOR = 0.53, 95% CI = 0.32, 0.87) lower odds of fistula repair success than those without one. Lastly, regarding fistula surgical repair complexity, the intermediate fistula repairs showed 64% (AOR = 0.36, 95% CI = 0.18, 0.70), and those rated difficult had 90% (AOR = 0.10, 95% CI = 0.05, 0.21) lower odds of success than simple repairs.

After running the full logistic regression model, we examined the Adjusted Marginal Probabilities (AMP) for all the statistically significant results as a post-estimation strategy to assess the effect sizes. Marginal probabilities give the expected probability of an outcome occurring for different levels or values of an independent variable, averaged across the distribution of all other variables in the model [29]. Table 3 presents all the AMPs, and below, we highlight the statistically significant results, where the 95% CIs do not include 1. For instance, the results showed that the patients from Northern and Muchinga provinces had a lower probability of having a successful fistula repair (AMP = 0.85; 95% CI = 0.82, 0.88) than those from Central and Lusaka (AMP = 0.91; 95% CI = 0.88, 0.95). The patients with a previous fistula repair had a lower probability of having a successful repair (AMP = 0.83; 95% CI = 0.78, 0.88) than those who did not have any previous fistula repairs (AMP = 0.89; 95% CI = 0.87, 0.91). Finally, those whose surgical repairs were intermediate (AMP = 0.90; 95% CI = 0.87, 0.90) or difficult (AMP = 0.74; 95% CI = 0.68, 0.80) had lower probabilities of success than those which were classified as simple (AMP = 0.95; 95% CI = 0.93, 0.98).

**Table 3:** Adjusted Marginal Probabilities (AMP) of successful obstetric fistula repair outcome for the associations in the fully adjusted model

**Table 3, part 1**	**AMP (95% CI)**	**Table 3, part 2**	**AMP (95% CI)**
**Socio-demographic factors**		**Fistula Factors continue**	
**Age group**		**Previous fistula repair**	
< 20	0.86 (0.79, 0.94)	No	0.89 (0.87, 0.91)
20–34	0.89 (0.87, 0.92)	Yes	0.83 (0.78, 0.88)
** ≥ 35**	0.86 (0.83. 0.89)	**Years lived with fistula**	
**Married**		< 1 year	0.91 (0.87, 0.94)
Not married	0.87 (0.83, 0.90)	1–5 years	0.86 (0.83, 0.89)
Married	0.89 (0.87, 0.91)	> 5 years	0.87 (0.85, 0.90)
Unknown	0.84 (0.78, 0.90)	**Surgery type**	
**Parity level**		Others	0.95 (0.90, 1.01)
**0**	0.75 (0.50, 0.99)	VVF repairs	0.86 (0.82, 0.90)
**1–3**	0.87 (0.85, 0.89)	**Healthcare Factors**	
** ≥ 4**	0.89 (0.86, 0.92)	**Hospital of care**	
**Province**		Monze Mission & Chilenje Level 1	0.88 (0.81, 0.95)
Central & Lusaka	0.91 (0.88, 0.95)	Kabwe Central & Solwezi General	0.84 (0.65, 1.03)
Copperbelt & N/Western	0.96 (0.91, 1.00)	Northern region hospitals (Mansa, Mbala & Chilonga)	0.87 (0.85, 0.90)
Eastern	0.87 (0.80, 0.93)	St. Francis Mission	0.90 (0.85, 0.95)
Luapula	0.86 (0.82, 0.90)	Unknown	0.76 (0.55, 0.96)
Northern & Muchinga	0.85 (0.82, 0.88)	**Surgery difficulty**	
Southern & Western	0.96 (0.90, 1.03)	Simple	0.95 (0.93, 0.98)
Unknown	0.89 (0.82, 0.97)	Intermediate	0.90 (0.87, 0.92)
**Fistula Factors**		Difficult	0.74 (0.68, 0.80)
**Fistula type**		**Post-complication**	
Rectovaginal Fistula (RVF) or both (RVF + VVF)	0.80 (0.61, 0.99)	None	0.88 (0.86, 0.90)
Vesicovaginal (VVF)	0.88 (86, 0.90)	Yes	0.84 (0.76, 0.92)
**Fistula classification**			
Type I	0.89 (0.86, 0.92)		
Type II A(a)	0.90 (0.87, 0.94)		
Type II A(b)	0.84 (0.76, 0.92)		
Type II B(a)	0.84 (0.80, 0.89)		
Type II B(b)	0.85 (0.78, 0.92)		
Type III	0.88 (0.83, 0.92)		

Note: The table continues at the top right

## Discussion

Our study revealed a pooled fistula repair success rate of 88.1%**,** strongly suggesting the effectiveness of surgery in managing obstetric fistulas and alleviating suffering among women who develop fistulas [14]. We identified the factors associated with successful obstetric fistula surgical repairs, including geographic location, previous surgical repair history, and fistula surgical repair complexity. Our findings provide valuable scientific insights into the benefits of optimizing obstetric fistula timely repairs and equitable resource allocation across the country's regions. Geographically, women from the Northern region of Zambia, including Northern and Muchinga provinces, which are generally rural and poor [30], showed significantly worse repair outcomes than women from the Central and Lusaka provinces. This result is cardinal in understanding the role of equitable health resource distribution in achieving Universal Health Coverage (UHC) in resource-limited settings [31]. Our study result highlighting poorer fistula repair outcomes in the rural Northern region of Zambia underscores the persistent disparities in hospital distribution, shortage of FIGO-trained fistula surgeons, medical supply stock-outs, limited access to accurate information, and delayed life-saving surgeries [21, 22, 25, 30].

Among fistula-related factors, our study revealed that a history of previous fistula repair was significantly associated with poorer outcomes. Prior evidence also showed that women undergoing repeat repairs were more likely to experience surgical failure due to the complexities introduced by scar tissue and reduced tissue integrity from prior surgeries [19, 21, 22]. Based on our study findings, obstetric fistula patients in Zambia face delays in accessing quality surgical care, such that most of them are treated by visiting surgeons in contexts where comprehensive treatment might not be feasible due to time constraints [5]. This is likely to lead to incomplete or sub-optimal surgical care and subsequent need for more complex future surgical repairs. FIGO, the Fistula Foundation Treatment Network, Ministries of Health, and partners like UNFPA employ a master surgeon training model to expand national capacity for fistula repair, effectively reducing the backlog of patients awaiting surgery [5, 32]. However, there is no information on the quality of these onsite capacity-building trainings, and no evidence exists to attribute failed fistula repairs to poor surgeon skills in Zambia. Our findings further underscore the urgent need to train more surgeons from all the country's provinces to integrate fistula management into the routine healthcare services that are readily available to those who require them. This will maximize success rates in primary surgical repairs, thereby avoiding multiple attempts on one patient [7, 15]. Additionally, addressing barriers to timely obstetric fistula treatment requires tackling stigma, shame, depression, financial constraints, transportation challenges, gender power imbalances, limited repair facilities, social reintegration struggles, and political leadership priorities that delay care [5].

Finally, our study results showed that women undergoing more complex and difficult fistula surgical repairs had lower success rates than those with simpler cases. This finding implies that the technical complexity and the risks associated with complications involved in more difficult fistula surgical repairs require advanced surgeon experience, which is scarce in Zambia [16, 19, 30]. The finding further emphasizes the need for establishing more specialized fistula repair centers across the country, resourced with highly skilled surgical teams to manage more complex cases, given that most of the surgeries analyzed in our study were conducted by visiting surgeons during fistula camps or outreaches [18].

### Strengths and limitations

Our study was based on a relevant theoretical framework and methodological approaches that made conclusions based on multiple variables–socio-demographic, fistula information, and healthcare factors–in the analysis, broadening the explanatory spectrum of the factors associated with successful obstetric fistula repair outcomes in Zambia. Additionally, we analyzed a large clinical dataset, which, to the best of our knowledge, was the first of its kind in Zambia, to draw our conclusions.

Nevertheless, we acknowledge that some potential confounders were not available in the Fistula Foundation Treatment Network dataset, which could bias the findings. For instance, the dataset did not have information on education level, employment status, socioeconomic status, duration of the fistula-associated labor, and maternal co-morbidities, such as HIV status and diabetes mellitus, that could be included in the analysis. Lastly, our assessment was limited to the analysis of factors measured up to the time when the patients were discharged from the hospitals. Therefore, we recommend more longitudinal studies that could assess factors measured at different points after discharge from the treatment centers, as these might reveal more nuanced findings over time.

## Conclusions and public health implications

Our study identified the factors associated with successful obstetric fistula repair outcomes, including geographic location, previous repair history, and surgical complexity. Obstetric fistulas are preventable by improved access to antenatal and comprehensive emergency obstetric care and can be treated by timely surgery. While substantial concerted efforts have been instituted to improve access to high-quality maternal and obstetric services, women who develop obstetric fistulas still experience barriers in accessing timely surgical repairs in most rural settings. Our study findings suggest that early diagnosis and increasing access to specialized surgical care by training more surgeons and setting more treatment centers close to the communities can significantly improve repair outcomes for women affected by obstetric fistula in resource-limited settings. Future efforts should focus on scaling the Fistula Foundation Treatment Network strategies to ensure equitable access to quality fistula care across different regions.

## Data Availability

The data that support the findings of this study are available from the Fistula Foundation Treatment Network in Zambia, but restrictions apply to the availability of these data, which were used under license for the current study and so are not publicly available. Data are, however, available from the authors upon reasonable request and with permission of the Fistula Foundation Treatment Network in Zambia.

## References

[1] Rushwan H, Khaddaj S, Knight L, Scott R. Need for a global obstetric fistula training strategy. Intl J Gynecology & Obste [Internet]. 2012 Oct [cited 2024 Oct 13];119(S1). Available from: https://obgyn.onlinelibrary.wiley.com/doi/10.1016/j.ijgo.2012.03.022 10.1016/j.ijgo.2012.03.022 22884819

[2] Wall LL. Obstetric vesicovaginal fistula as an international public-health problem. The Lancet. 2006;368(9542):1201–9. 10.1016/S0140-6736(06)69476-2 17011947

[3] Wall LL. Preventing Obstetric Fistulas in Low-Resource Countries. Obstet Gynecol Surv. 2012;67(2):111–21. 22325301 10.1097/OGX.0b013e3182438788

[4] Ramphal S, Moodley J. Vesicovaginal fistula: obstetric causes. Curr Opin Obstet Gynecol. 2006;18(2):147–51. 16601475 10.1097/01.gco.0000192980.92223.2d

[5] Baker Z, Bellows B, Bach R, Warren C. Barriers to obstetric fistula treatment in low-income countries: a systematic review. Tropical Med Int Health. 2017;22(8):938–59. 10.1111/tmi.12893 28510988

[6] Waaldijk K, Armiya’u YD. The obstetric fistula: A major public health problem still unsolved. International Urogynecology Journal. 1993 Mar;4(2):126–8.

[7] Rushwan H, Khaddaj S, Knight L, Scott R. Need for a global obstetric fistula training strategy. International Journal of Gynecology and Obstetrics. 2012;119(SUPPL.1). 10.1016/j.ijgo.2012.03.022 22884819

[8] Cowgill KD, Bishop J, Norgaard AK, Rubens CE, Gravett MG. Obstetric fistula in low-resource countries: an under-valued and under-studied problem – systematic review of its incidence, prevalence, and association with stillbirth. BMC Pregnancy Childbirth. 2015;15(1):193. 26306705 10.1186/s12884-015-0592-2 PMC4550077

[9] Slinger G, Trautvetter L. Addressing the fistula treatment gap and rising to the 2030 challenge. Int J Gynecol Obstet. 2020;148(S1):9–15. 10.1002/ijgo.13033 PMC7004198 31943185

[10] Swain D, Parida S, Jena S, Das M, Das H. Obstetric fistula: A challenge to public health. Indian J Public Health. 2019;63(1):73. 30880741 10.4103/ijph.IJPH_2_18

[11] Kyomuhendo GB. Low Use of Rural Maternity Services in Uganda: Impact of Women’s Status, Traditional Beliefs and Limited Resources. Reprod Health Matters. 2003;11(21):16–26. 12800700 10.1016/s0968-8080(03)02176-1

[12] Adler AJ, Ronsmans C, Calvert C, Filippi V. Estimating the prevalence of obstetric fistula: a systematic review and meta-analysis. BMC Pregnancy Childbirth. 2013;13(1):246. 24373152 10.1186/1471-2393-13-246 PMC3937166

[13] Pollaczek L, Rajagopal K, Chu J. Patient characteristics, surgery outcomes, presumed aetiology and other characteristics of fistula surgeries and related procedures supported by Fistula Foundation from 2019 to 2021: a multicentre, retrospective observational study. BMJ Open. 2024;14(3): e078426. 38485171 10.1136/bmjopen-2023-078426 PMC10941128

[14] Higashi H, Barendregt J, Kassebaum N, Weiser T, Bickler S, Vos T. Surgically avertable burden of obstetric conditions in low- and middle-income regions: a modelled analysis. BJOG. 2015;122(2):228–36. 25546047 10.1111/1471-0528.13198

[15] Meara JG, Leather AJM, Hagander L, Alkire BC, Alonso N, Ameh EA, et al. Global Surgery 2030: evidence and solutions for achieving health, welfare, and economic development. Int J Obstet Anesth. 2016;25:75–8. 26597405 10.1016/j.ijoa.2015.09.006

[16] Zeleke LB, Welsh A, Abeje G, Khajehei M. Treatment outcomes of obstetrical fistula surgical repair in low‐ and middle‐income countries: A scoping review. Intl J Gynecology & Obste. 2024 Jun 16;ijgo.15724. 10.1002/ijgo.15724 38881203

[17] Swain D, Parida SP, Jena SK, Das M, Das H. Prevalence and risk factors of obstetric fistula: implementation of a need-based preventive action plan in a South-eastern rural community of India. BMC Women’s Health. 2020;20(1):40. 32131799 10.1186/s12905-020-00906-w PMC7055058

[18] Obstetric Fistula Strategic Plan 2022 2026 Final.

[19] Bernard L, Giles A, Fabiano S, Giles S, Hudgins S, Olson A, et al. Predictors of Obstetric Fistula Repair Outcomes in Lubango, Angola. J Obstet Gynaecol Can. 2019;41(12):1726–33. 30987849 10.1016/j.jogc.2019.01.025

[20] Voskamp T, Khisa WW, Oosting RM, Wiggers T, Dankelman J. A training phantom for a vesicovaginal fistula repair with the transvaginal approach. Curr Probl Surg. 2024;61(8): 101550. 39098338 10.1016/j.cpsurg.2024.101550

[21] Hareru HE, Ashuro Z, Debela BG, Abebe M. Obstetric fistula repair failure and its associated factors among women who underwent repair in sub-Saharan Africa. A systematic review and meta-analysis. Chauke HL, editor. PLoS ONE. 2024 Feb 5;19(2):e0295000. 10.1371/journal.pone.0295000 PMC10843137 38315695

[22] Imakando MM, Michelo C, Mkandawire T, Kasonka L. Characteristics and Surgical Repair Outcomes of Obstetric Fistula Patients Managed at a Teaching Hospital in Zambia: A Retrospective Cross Sectional Study. MJZ. 2022;49(2):146–56.

[23] Tanahashi T. Health service coverage and its evaluation. Bull World Health Organ. 1978;56(2):295–303. 96953 PMC2395571

[24] Bai J, Wong FWS, Bauman A, Mohsin M. Parity and pregnancy outcomes. Am J Obstet Gynecol. 2002;186(2):274–8. 11854649 10.1067/mob.2002.119639

[25] Tembo NN, Maluwa A, Odland J, Mukwato PK, Kwaleyela CN, Zulu JM, et al. Barriers in seeking fistula repair services among women with obstetric fistula in Zambia: The case of Muchinga, Luapula Eastern and Southern Provinces. Int J Midwifery Nurs Pract. 2020;3(1):13–20.

[26] Zambia Statistics Agency M of H (MOH) Z and I. Zambia demographic and health survey 2018. Lusaka, Zambia, and Rockville, Maryland, USA; 2018.

[27] Ryoo SB, Oh HK, Ha HK, Han EC, Kwon YH, Song I, et al. Outcomes of surgical treatments for rectovaginal fistula and prognostic factors for successful closure: a single-center tertiary hospital experiences. Ann Surg Treat Res. 2019;97(3):149. 31508396 10.4174/astr.2019.97.3.149 PMC6722290

[28] Royston P, White I. Multiple Imputation by Chained Equations (MICE): Implementation in *Stata*. J Stat Soft [Internet]. 2011 [cited 2024 Oct 14];45(4). Available from: http://www.jstatsoft.org/v45/i04/

[29] Shaw BP. Effect sizes for contrasts of estimated marginal effects. Stand Genomic Sci. 2022;22(1):134–57.

[30] Moraes AN, Likwa RN, Nzala SH. A retrospective analysis of adverse obstetric and perinatal outcomes in adolescent pregnancy: the case of Luapula Province, Zambia. Maternal Health, Neonatology and Perinatology. 2018;4(1):20. 30349732 10.1186/s40748-018-0088-y PMC6192102

[31] Pandey KR. From health for all to universal health coverage: Alma Ata is still relevant. Global Health. 2018;14(1):62. 29970118 10.1186/s12992-018-0381-6 PMC6029383

[32] Chin EA, Arrowsmith S. Training and capacity building in obstetric fistula repair: A scoping review. Intl J Gynecology & Obste. 2024;164(1):11–8. 10.1002/ijgo.14901 37306124

